# A universal bioluminescence resonance energy transfer sensor design enables high-sensitivity screening of GPCR activation dynamics

**DOI:** 10.1038/s42003-018-0072-0

**Published:** 2018-08-07

**Authors:** Hannes Schihada, Sylvie Vandenabeele, Ulrike Zabel, Monika Frank, Martin J. Lohse, Isabella Maiellaro

**Affiliations:** 1 Institute of Pharmacology and Toxicology and Rudolf Virchow Center, University of Würzburg, Würzburg, Germany; 2 Max Delbrück Center for Molecular in Medicine, Berlin, Germany

## Abstract

G-protein-coupled receptors (GPCRs) represent one of the most important classes of drug targets. The discovery of new GCPR therapeutics would greatly benefit from the development of a generalizable high-throughput assay to directly monitor their activation or de-activation. Here we screened a variety of labels inserted into the third intracellular loop and the C-terminus of the α_2A_-adrenergic receptor and used fluorescence (FRET) and bioluminescence resonance energy transfer (BRET) to monitor ligand-binding and activation dynamics. We then developed a universal intramolecular BRET receptor sensor design to quantify efficacy and potency of GPCR ligands in intact cells and real time. We demonstrate the transferability of the sensor design by cloning β_2_-adrenergic and PTH1-receptor BRET sensors and monitored their efficacy and potency. For all biosensors, the Z factors were well above 0.5 showing the suitability of such design for microtiter plate assays. This technology will aid the identification of novel types of GPCR ligands.

## Abstract (web summary)

Hannes Schihada et al. report the design of 11 BRET-based biosensors that allow for quantification of GPCR ligand-binding dynamics in a micro-titer format. The biosensors achieve higher dynamic range and sensitivity than FRET-based biosensors and their design can be extended to the study of other receptor types.

## Introduction

G-protein-coupled receptors (GPCRs) constitute the largest and the most diverse group of membrane receptors in eukaryotes. They play a role in a plethora of cellular processes. Despite their functional diversity, they share a similar molecular architecture of seven transmembrane helices and a conserved mechanism of activation. Binding of agonist ligands to their cognate receptor leads to a change in the arrangement of distinct transmembrane helices, and a pronounced outward movement of helix 6 (up to 14 Å)^1^, which is then propagated to the third intracellular loop that connects helix 5 with 6, thus enabling the engagement of several downstream signaling pathways, most importantly the activation of G-proteins. We previously demonstrated that such ligand-induced conformational changes can be visualized in living cells by Förster Resonance Energy Transfer (FRET). The average distance between the third intracellular loop and the C-terminus of receptors (e.g., β_2_AR 6.2 nm)^2^ is within the range addressable by FRET (2.4–7.2 nm)^3^. Therefore, tagging these conformationally sensitive sites with fluorescent donors and acceptors permits the recording of receptor activation as a change in energy transfer between these chromophores. Following this principle, a series of intramolecular FRET-based GPCR-biosensors have been generated, notably for the α_2A_-adrenergic receptor (α_2A_AR), employing the fluorescent proteins CFP and YFP (α_2A_AR_CFP/YFP_)^4,5^ or CFP with the small Fluorescein Arsenical Hairpin Binder (FlAsH) as fluorophores^6^.

These FRET-based GPCR biosensors have become widely used tools^7^ and represent the most direct unbiased way to determine the effects of ligands on a given receptor. Their employment has helped to elucidate several aspects of receptor pharmacology and kinetics^8^. However, the combination of donor and acceptor fluorophores used so far suffers from a low signal-to-noise ratio and high fluorescence background which limits their use to single-cell experiments, slowing down the characterization of new pharmacological compounds as GPCR-directed therapeutics. More recently, Bioluminescence Resonance Energy Transfer (BRET) has been tested as an alternative approach to monitor the conformational change of receptors. BRET occurs between proximally situated donor–acceptor pairs (1.6–8.5 nm)^9^, but here a light emitting enzyme luciferase is used as a donor, sidestepping many of the deficiencies associated with direct illumination of the sample. Earlier attempts to generate intramolecular GPCR BRET-based biosensors employing Renilla Luciferase as a donor in combination with GFP or FlAsH as acceptor showed poor agonist-induced BRET changes^10–13^.

Despite the many efforts in optimizing the way in which we study GPCRs and a plethora of methods to assess ligand binding or downstream signaling, the lack of a generalizable assay to monitor directly in living cells receptor activation or de-activation in a format suitable for high-throughput screening is slowing down the progress in discovering new GPCR therapeutics. In the present study, we therefore set out to permutate the well-characterized FRET-based biosensor α_2A_AR_CFP/YFP_ in order to develop a universal and versatile sensor design to reach the stringent requirements of monitoring receptor conformational dynamics in intact cells in microtiter plates.

Preserving the fluorophore insertion sites in the third intracellular loop and C- terminus, we investigated whether combining different donors and acceptors would improve the transfer of energy to enable the visualization of the receptor’s conformational changes in microtiter plates. Among the 10 FRET-based and the 11 BRET-based α_2A_AR biosensors generated in this study, the highest amplitude in the signals upon norepinephrine stimulation was recorded with the BRET-based α_2A_AR biosensors combining NanoLuc^14^ luciferase as the donor and the self-labeling protein tag Halo labeled with the HaloTag dye NanoBRET 618 as the acceptor. The EC_50_-values calculated for these BRET experiments were in line with binding affinities, demonstrating that the developed BRET biosensor α_2A_AR_Nluc/Halo_ assay faithfully reports ligand affinities. This feature was conserved also for a β_2_-adrenergic and a PTH1 receptor BRET-based biosensors. Also for these two receptors, the agonist-induced BRET changes were in line with the binding data suggesting that our receptor biosensor design is a generalizable design that may be use in lieu of the endogenous receptor to determine efficacy and potency of ligands. To assess the applicability of the biosensor design to microtiter plate and its scalability, we performed for each receptor a Z-factor analysis^15^ to quantify the quality and reproducibility of the assays. For all receptors analyzed, the Z-factor was well above 0.5—which characterizes an excellent assay suitable to be used in microtiter plates. We propose that this technology will speed the characterization of new pharmacological compounds acting at GPCRs.

## Results

### Dynamic range of FRET- and BRET-based α_2A_AR biosensors

**Fig. 1:**
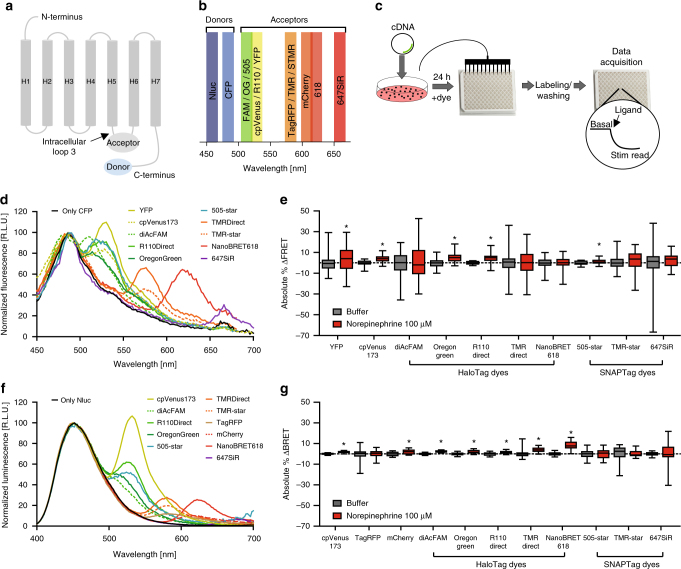
Evaluation of intramolecular FRET and BRET α_2A_-adrenergic receptor biosensors. **a** Schematic of the biosensor. **b** Emission peaks of chromophores. **c** Workflow. **d** FRET emission spectra of the CFP-label alone or with fluorescent acceptors (each *N* = 1). **e** FRET changes (%) induced by norepinephrine normalized for buffer (YFP: *N* = 7, cpVenus173: *N* = 4, diAcFAM: *N* = 6, Oregon Green: *N* = 5, R110 Direct: *N* = 3,TMR Direct: *N* = 6, NanoBRET 618: *N* = 6, 505-star: *N* = 5, TMR-star: *N* = 5, 647SiR: *N* = 4). **f** BRET emission spectra of the NanoLuc-label alone or with fluorescent acceptors (each *N* = 1). **g** BRET changes (%) induced by norepinephrine normalized for buffer (cpVenus173: *N* = 7, TagRFP: *N* = 4, mCherry: *N* = 4, diAcFAM: *N* = 3, Oregon Green: *N* = 3, R110 Direct: *N* = 3, TMR Direct: *N* = 3, NanoBRET 618: *N* = 7, 505-star: *N* = 3, TMR-star: *N* = 5, 647SiR: *N* = 5). Data in **e** and **g** show box and whisker plots. Difference was analyzed by two-way ANOVA followed by Bonferroni post hoc test. *p ≤ 0.05 vs. buffer

With the goal of generating a GPCR biosensor design to monitor receptor activation in microtiter plates, we permutated the well-characterized FRET-based biosensor α_2A_AR_CFP/YFP_. We preserved the fluorophore insertion sites in the third intracellular loop and C-terminus (Fig. 1a) but we substituted the original YFP with its brighter variant cpVenus or with self-labeling protein tags: *SNAP* (α_2A_AR_CFP/SNAP_; 20 kDa)^16^ or *Halo* (α_2A_AR_CFP/Halo_; 36 kDa)^17^. Both of these tags can be labeled covalently with exogenous cell-permeable fluorescent dyes. Their diverse emission and excitation peaks (Fig. 1b, Supplementary Fig. 1), degrees of spectral overlap with the donor emission (CFP) and photophysical properties impacted the FRET behavior that we observed, allowing us to optimize our approach using a modular fluorophore within the receptor chassis. In this optimization phase, HEK cells were transiently transfected with the different α_2A_AR biosensors and, after specific labeling, experiments were performed in 96-well plates (Fig. 1c). Each FRET-based pair exhibited a different degree of basal transfer of energy—as demonstrated by their respective FRET emission spectra (Fig. 1d). However, only five of the ten FRET-based pairs tested showed a detectable agonist-induced change in FRET (ΔFRET%) that did not exceed 5% when stimulated by the full agonist norepinephrine (100 µM) (Fig. 1e).

With the goal of obtaining a higher dynamic range, we tested BRET as possibly more sensitive approach. Here, we used the relatively small-sized NanoLuc^14^ luciferase (19 kDa; Nluc) that possesses, in presence of its substrate (furimazine), a narrow bioluminescence spectrum, high brightness, and physical stability.

We generated 11 BRET-based α_2A_AR biosensors combining the donor Nluc with fluorescent proteins of different color from yellow (cpVenus) to orange-red (mCherry; TagRFP), or with the self-labeling Halo or SNAP tags. Applying the workflow developed above (Fig. 1c), we tested the ability of the α_2A_AR BRET biosensors to report effects of the full agonist norepinephrine in microtiter plate format. All the tested receptor BRET-sensors exhibited a basal energy transfer (Fig. 1f), while the activation of the receptor induced by norepinephrine at a concentration of 100 μM was detectable in seven of them. The highest amplitude was recorded for Nluc combined with the HaloTag dye NanoBRET 618^18^ (α_2A_-AR_Nluc/Halo**618**_; ΔBRET % = 8.15 ± 0.72) (Fig. 1g)—about two-fold higher than all other 20 FRET- and BRET-biosensors tested. No further improvement in the amplitude of ΔBRET was obtained by swapping the positions of donor and acceptor (Supplementary Fig. 2).

### Pharmacology of the α_2A_-AR_Nluc/Halo618_ BRET-based biosensor

**Fig. 2:**
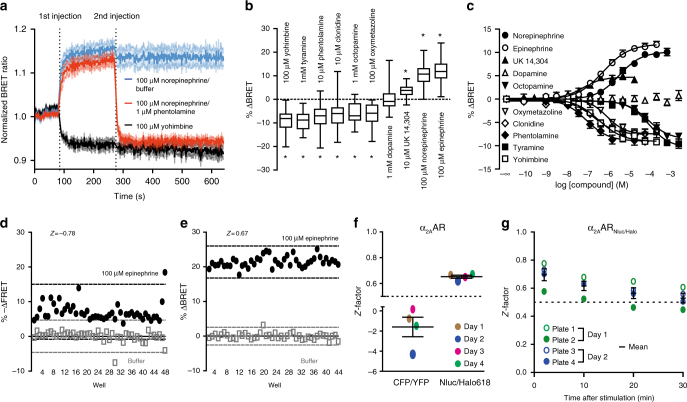
Pharmacological characterization of the α_2A_AR_Nluc/Halo618_ BRET-based biosensor, and evaluation of its applicability for microtiter plate screening. **a** Time-course of the normalized BRET ratio upon ligand stimulation (each *N* = 3). **b** Ligand-induced maximal BRET changes (Yohimbine: *N* = 7, Tyramine: *N* = 10, Phentolamine: *N* = 11, Clonidine: *N* = 10, Octopmaine: *N* = 5, Oxymetazoline: *N* = 6, Dopamine: *N* = 6, UK 14,304: *N* = 10, Norepinephrine: *N* = 25, Epinephrine: *N* = 23). **c** Concentration–response curves of different α_2A_AR ligands (Yohimbine: *N* = 7, Tyramine: *N* = 4, Phentolamine: *N* = 5, Clonidine: *N* = 4, Oxymetazoline: *N* = 4, Octopamine: *N* = 4, Dopamine: *N* = 4, UK 14,304: *N* = 3, Norepinephrine: *N* = 6,Epinephrine: *N* = 4). Comparison of the Z-factors for cells expressing the FRET_CFP/YFP_ or BRET_Nluc/Halo_ sensor of α_2A_AR. **d** FRET or **e** BRET changes 2 min after 100 μM epinephrine or buffer stimulation are plotted for each well of representative plates and **f** the average values (each *N* = 4). **g** Z-factor at different time points after addition of 100 μM epinephrine or buffer (*N* = 4). Data show box and whisker plots (**b**) or mean ± s.e.m (**a**, **c**, **f**, **g**). Difference was analyzed by two-way ANOVA followed by Bonferroni post hoc test. *p ≤ 0.05 vs. buffer

We then sought to ascertain that the α_2A_AR_Nluc/Halo**618**_ faithfully recorded efficacies and potencies of a panel of known α_2A_AR ligands (Supplementary Table 1). We created a cell line stably expressing this biosensor, and after optimizing the assay conditions (Supplementary Fig. 3), we measured ligand-induced BRET-responses. Stimulation with the full agonist norepinephrine (100 μM) and the inverse agonist yohimbine (100 µM) induced changes in BRET with opposite directions (Fig. 2a; agonist: positive ΔBRET blue line; inverse agonist: negative ΔBRET black line)—mirroring the opposite pharmacological effect exerted at the receptor level. The changes in BRET evoked by both ligands were fast, reaching a plateau 120 s after their injection, and remained stable for more than 30 min (Supplementary Fig. 4). The injection of the antagonist phentolamine (1 µM) reverted the effect of norepinephrine (Fig. 2a; red line), demonstrating the applicability of the biosensor to monitor inactivation kinetics. A panel of seven additional ligands triggered responses ranging between the two extremes, norepinephrine and yohimbine, and compatible with their known efficacies as full, partial, or inverse agonists (Fig. 2b, Supplementary Table 1). Full concentration–response curves were performed for all ten compounds (Fig. 2c). Radioligand binding experiments demonstrated that the BRET biosensor α_2A_AR_Nluc/Halo_ had binding affinities (pK_i_-values) similar to the wild-type receptor **(**Supplementary Table 2**)**, and they correlate with the measured EC_50_-values **(**Supplementary Table 3**)**, indicating that our assay faithfully reports ligand affinities. Interestingly, for agonists these EC_50_-values were similar to the high-affinity component of the competition curves (pK_H_) generally assumed to represent the receptor/G-protein complex^19^. This indicates some constitutive activity of the α_2A_AR_Nluc/Halo_ biosensor and its interaction with endogenous G-proteins. The relative BRET-change induced by neutral ligands and also the characterization of G-protein activation (Supplementary Fig. 5) demonstrated some constitutive activity of the biosensor. Such constitutive activity, not seen with analogous GFP-based sensors^20^, might be due to the larger size of the tags employed that might separate helices 5 and 6, which would shift the receptor to a more active state. However, this effect did not change the expected order of efficacies of the various ligands and even facilitated the characterization of both, inverse agonists and antagonists. Overall, these data indicate that the intramolecular α_2A_AR_Nluc/Halo**618**_ BRET-biosensor faithfully reports the activation state of the α_2A_AR in 96-well plates.

### Microtiter plate suitability of the α_2A_AR_Nluc/Halo618_

To validate the suitability of the novel BRET-based biosensor α_2A_AR_Nluc/Halo**618**_ to monitor receptor conformation in microtiter plate format, we performed the Z-factor analysis^15^ to quantify the quality and reproducibility of the assay. In this analysis, values *Z* < 0 defines unusable assays, *Z* = 1 approximates a “perfect” assay, and *Z* ≥ 0.5 characterizes an excellent assay. Compared to the original α_2A_AR_CFP/YFP_, classified as unusable (*Z* = –1.60 ± 0.97; Fig. 2d, f), α_2A_AR_Nluc/Halo**618**_ yielded an excellent assay (*Z* = 0.65 ± 0.01; Fig. 2e, f) that was stable for more than 20 min (Fig. 2g).

### β_2_-adrenergic receptor BRET-biosensor (β_2_AR_Nluc/Halo618_)

**Fig. 3:**
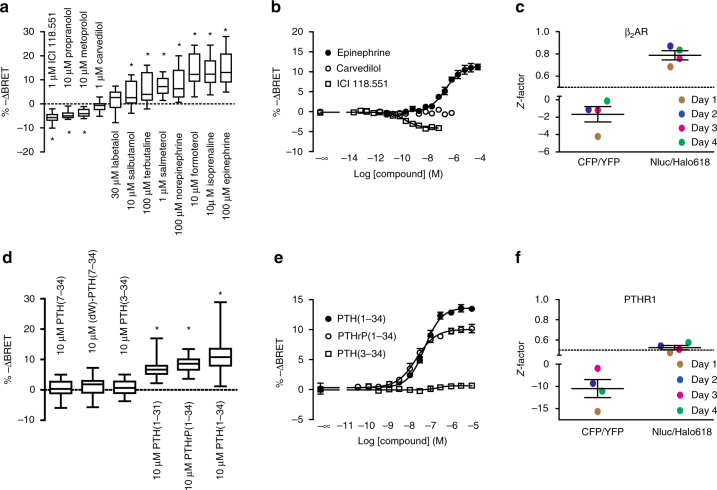
Transferability and microtiter plate applicability of the new intramolecular receptor-biosensor design. BRET changes reported by β_2_AR_Nluc/Halo618_ expressing cells upon (**a**) saturating ligand stimulation (ICI 118.551: *N* = 6, Propranolol: *N* = 6, Metoprolol: *N* = 6, Carvedilol: *N* = 12, Labetalol: *N* = 16, Salbutamol: *N* = 8, Terbutaline: *N* = 8, Salmeterol: *N* = 11, Norepinephrine: *N* = 8, Formoterol: *N* = 11, Isoprenaline: *N* = 21, Epinephrine: *N* = 21) and **b** serial ligand dilutions to obtain concentration–response curves (Epnephrine: *N* = 4, Carvedilol: *N* = 4, ICI 118.551: *N* = 6). **c** Mean of the *Z*-factor of β_2_AR_CFP/YFP_ and β_2_AR_Nluc/Halo618_ (each *N* = 4). BRET changes reported by PTHR1_Nluc/Halo618_ expressing cells upon (**d**) saturating ligand stimulation (PTH(7-34): *N* = 12, (dW)-PTH(7-34): *N* = 12, PTH(3-34): *N* = 14, PTH(1-31): *N* = 6, PTHrP(1-34): *N* = 14,PTH(1-34): *N* = 22) and **e** serial ligand dilutions to obtain concentration–response curves (PTH(1-34): *N* = 3, PTHrP(1-34): *N* = 3, PTH(3-34): *N* = 6). **f** Mean of the *Z*-factor of PTHR1_CFP/YFP_ and PTHR1_Nluc/Halo618_ (each *N* = 4). Data are expressed as box and whisker plots (**a**, **d**) or mean ± s.e.m (**b**, **c**, **e**, **f**). Difference was analyzed by two-way ANOVA followed by Bonferroni post hoc test. *p ≤ 0.05 vs. buffer

To demonstrate the transferability of the BRET_Nluc/Halo**618**_ as a general design to pharmacologically profile any GPCR, we generated an analogous β_2_-adrenergic receptor biosensor (β_2_AR_Nluc/Halo**618**_) and measured efficacy and potency of different ligands in microtiter plate format. The β_2_AR_Nluc/Halo**618**_ biosensor showed signaling activity similar to the wild-type receptor (Supplementary Fig. 6a–c). Full agonist stimulation induced a change in BRET opposite to that of an inverse agonist, while the amplitudes for antagonists and partial agonists were intermediate (Fig. 3a), demonstrating that the ability of reporting the efficacy of ligands was preserved. The EC_50_-values obtained from concentration–response curves for the full agonist epinephrine, the inverse agonist ICI 118,551 and the neutral antagonist carvedilol (Fig. 3b), were similar to data obtained by radioligand binding assay (Supplementary Table 4), demonstrating the β_2_AR BRET-based biosensor was reporting wild-type affinity and efficacy. Interestingly, we found that norepinephrine, usually considered a full agonist when monitored at the second messenger level^21^ (cAMP), induced only a partial conformational change of β_2_AR_Nluc/Halo**618**_—in line with other conformational studies^22,23^. Our data would also support the new evidence suggesting that UK 14,304 behaves as a partial^24^ more than a full agonist. This demonstrates that BRET-based biosensors are a powerful unbiased approach, since they bypass the effect of signal amplification and receptor reserve. Again, the *Z*-factor of ≈ 0.8 was greatly improved compared to the earlier FRET β_2_AR-biosensor and indicated the suitability for high-throughput screening (Fig. 3c; Supplementary Fig. 8)

### Parathyroid hormone receptor 1 BRET-biosensor (PTHR1_Nluc/Halo618_)

To further substantiate the transferability of the BRET_Nluc/Halo**618**_ sensor design, we devised an analogous class B parathyroid hormone receptor 1 (PTHR1) BRET-biosensor (PTHR1_Nluc/Halo**618**_). Again, this biosensor retained signaling activity (Supplementary Fig. 6d–f), efficacy (Fig. 3d) and affinity (Fig. 3e) similar to the wild-type receptors (Supplementary Table 5) for the analyzed peptides.

Application of the full agonist PTH(1-34) evoked a ≈10% change in BRET. Antagonists had no detectable effects (Fig. 3d), but retained their ability of inhibiting receptor activation in competition experiments (Supplementary Fig. 7). In contrast to the α_2A_AR and β_2_AR, no inverse agonists are available for the PTHR1 that might cause effects opposite to those of PTH(1-34). The *Z*-factor for PTHR1_Nluc/Halo**618**_ (0.52 ± 0.02) demonstrated that also this BRET-biosensor was suitable for high-throughput screening (Fig. 3f; Supplementary Fig. 8).

## Discussion

Taken together, we have developed a universal sensor design for GPCRs that retains the signaling capacity of the receptors, resolves both efficacy and potency of ligands, and works in intact cells and in real time in a microtiter plate format. In a single assay, this approach offers the analysis of activation/de-activation kinetics, potency and direct efficacy–which makes it a rapid and high content screening approach. The throughput of this assay essentially depends on the equipment available for pipetting and plate reading. Under optimal conditions, it depends only on the time required to read the plate before and after addition of compounds, while under the most basic conditions, the time required for a single plate would encompass the times required for the basal read, addition of compounds, and a second read. If the labeling procedure is carried out in serum- and phenol red-free medium, then no washing step is required.

As any optical detection method, the readout of our BRET-based receptor biosensor may be impaired by molecules that interfere with the absorption properties; bind to the energy donor or acceptor interfering with their emission properties; and compete with the substrate, causing inhibition of the luciferase^25^. The chemical evaluation of compounds of a screening library can help discriminating false positives. Appropriate controls for these issues therefore need to be included in such a screening approach. An advantage of our receptor biosensor is that the tags are localized intracellularly, which limits their susceptibility to cell permeable compounds.

To the best of our knowledge, this generalizable design offers the first possibility to upscale the study of receptor activation and deactivation - which represents the most direct and unbiased way to estimate the effect of any chemical entity on receptors of interest, facilitating the discovery of new therapeutic compounds. These GPCR biosensors should prove useful in determining ligand properties at known GPCRs and in elucidating the binding properties of orphan receptors. Furthermore, their high sensitivity may allow their use to monitor GPCR activation in situ using new knock-in technologies^26^.

## Methods

### cDNA constructs

The FRET sensor α_2A_AR_CFP/YFP_^4^ was used as starting construct to generate the various α_2A_-adrenergic receptor FRET and BRET sensors (α_2A_AR_donor/acceptor_) described in this study. The donors (CFP or Nluc) were fused to Val461 at the C-terminus while all acceptors tested were placed in the third intracellular loop between Ala250 and Ser371. The BRET-based β_2_-adrenergic receptor biosensor β_2_AR_Nluc/Halo_ was cloned starting from the previously described FRET version^22^ introducing HaloTag in the third intracellular loop between Asp251 and Gly252 and Nluc in the C-terminus at Glu369. The BRET sensor for the parathyroid hormone receptor (PTHR1) was cloned starting from the previously described FRET version^4^. HaloTag was inserted in the third intracellular loop between Gly395 and Arg396 and Nluc was fused to Gly497 of the C terminus. All tag exchanges were performed employing established PCR strategies and restriction and ligation enzymes. Constructs were cloned into a pcDNA3 vector and verified by sequencing.

### Plasmids

cDNA encoding the fluorescent protein cpVenus^173^ was amplified from the Gα_i2_-sensor v2.0^27^. The SNAPtag sequence was amplified using a SNAPtag-GABA_B1_ template kindly provided by J.P. Pin (Institut de Génomique Fonctionnelle, Montpellier, France)^28^. Sequences encoding TagRFP (pTagRFP-C vector) and mCherry were purchased from Evrogen and Addgene, respectively. cDNA encoding HaloTag (pFC14K HaloTag® CMV Flexi® Vector) and NanoLuciferase (pFC32K Nluc CMV-neo Flexi® Vector) were purchased from Promega. Gα_i2_-FRET^27^ sensor was kindly provided by J. Goedhart (Section Molecular Cytology, University of Amsterdam, Amsterdam, The Netherlands), the H187-EPAC-FRET sensor^29^ was kindly provided by K. Jalink (The Netherlands Cancer Institute, Amsterdam, The Netherlands).

### Reagents

(−)-Epinephrine, l-(−)-norepinephrine (+)-bitartrate salt monohydrate, UK 14,304, dopamine hydrochloride, (±)-octopamine hydrochloride, clonidine hydrochloride, tyramine hydrochloride, phentolamine hydrochloride, yohimbine hydrochloride, isoprenaline hydrochloride, formoterol fumarate dihydrate, terbutaline hemisulfate salt, salmeterol xinofoate, salbutamol hemisulfate salt, metoprolol tartrate, (±)-propranolol hydrochloride, ICI 118,551 hydrochloride, labetalol hydrochloride, carvedilol, GTP, poly-D-lysine, G-418 and the fluorescent monoclonal antibody Anti-Flag® M2-Cy3 and Millipore glass-fiber filters for radioligand saturation binding were from Sigma-Aldrich. [^3^H]RX821002 was purchased from Hartmann Analytic. The HA-tag monoclonal antibody (16B12) Alexa Fluor 488 was from ThermoFisher Scientific. Oxymetazoline hydrochloride was purchased from Tocris. The peptide ligands PTH(1-34) (catalog number: H-4835), PTH(7-34) (catalog number: N-1110), (dw)-PTH(7-34) (catalog number: H-9115), PTHrP(1-34) (H-6630), PTH(1-31) (catalog number: H-3408) and PTH(3-34) (catalog number: H-3088) were from BACHEM. All HaloTag fluorescent dyes were purchased from Promega. SNAP fluorescent dyes were from NEB. White-wall, white bottomed and black-wall, black-bottomed 96-well plates were purchased from Brand. MultiScreen® Filter plates for radioligand competition binding were from Millipore.

### Cell culture and transfection

HEK-TSA cells used for transient expression of constructs, were grown in Dulbecco’s Modified Eagle’s Medium (DMEM) supplemented with 2 mM glutamine, 10% fetal calf serum, 0.1 mg mL^−1^ streptomycin, and 100 units per mL penicillin at 37 °C with 5% CO_2_. HEK293 cells were used for the development of stable BRET sensor cell lines. Cells grown in 100 mm dishes were transfected at a confluence of 50–70% with 5 µg of DNA using Effectene Transfection Reagent Kit (Qiagen) according to the manufacturer’s instructions. Transfected clones were selected with 600 μg mL^−1^ of G-418 and clonal lines were maintained in DMEM supplemented with 200 μg mL^−1^ G-418, 2 mM glutamine, 10% fetal calf serum, 0.1 mg mL^−1^ streptomycin, and 100 units per mL penicillin at 37 °C with 5% CO_2_.

### Transient transfection and plating

For transient expression of the sensors, 1.5 × 10^6^ HEK-TSA cells were seeded onto a 55 mm dish and transfected the day after with 2 µg of plasmids encoding the biosensors using Effectene Transfection Reagent (Qiagen) according to the manufacturer’s protocol. In case two different plasmids were co-transfected, 4 µg was used as total amount of DNA in a 1:1 ratio of the two plasmids. Twenty-four hours after transfection, cells were transferred to poly-d-lysine pre-coated black-wall, black-bottomed (FRET experiments) or white-wall, white-bottomed (BRET experiments) 96-well plates at a density of 50,000 (FRET) or 20,000 (BRET) cells per well.

### Fluorescence labeling of FRET and BRET acceptors

Labeling with all dyes was performed at 37 °C and 5% CO_2_ in 96-well plates. All dyes were dissolved in DMEM. HaloTag® diAcFAM (1 µM), HaloTag® Oregon Green® (1 µM), SNAP-cell 505-Star (10 µM), SNAP-cell TMR-Star (3 µM) and SNAP-cell 647SiR (3 µM) were incubated for 30 min 48 h after transfections. Excessive dye was washed out three times followed by incubation with fresh DMEM for additional 30 min (37 °C and 5% CO_2_). HaloTag® R110Direct, HaloTag® TMRDirect and HaloTag® NanoBRET 618 required overnight labeling at a concentration of 100 nM. A minimum of 4 wells remained unlabeled to serve as correction for donor bleedthrough (unlabeled control).

### Measurement of fluorescence excitation and emission spectra

HEK-TSA cells were transfected with different BRET based α_2A_AR biosensors and labeled as described above, without substrate, in order to read only the emission and excitation spectra of the different acceptors. YFP and CFP spectra were collected using the FRET α_2A_AR_CFP/Halo_ and α_2A_AR_YFP/Halo_ biosensors without HaloTag labeling. All spectra were measured in buffer (2 mM HEPES, 28 mM NaCl, 1.08 mM KCl, 0.2 mM MgCl_2_, 0.4 mM CaCl_2_, pH 7.3) with 2 nm resolution from 400 to 700 nm using a CLARIOstar plate reader (BMG). Spectra are expressed as a percentage of the respective maximal excitation or emission peak.

### Measurement of FRET and BRET emission spectra

HEK-TSA cells were transfected and labeled as described above. Emission spectra were recorded in buffer with 2 nm resolution from 400 to 700 nm upon donor excitation at 420 nm (FRET sensors) or addition of 1:1000 furimazine dilution (BRET) using a CLARIOstar plate reader (BMG). Spectra are expressed as a percentage of the maximal donor emission peak.

### FRET measurements

Cells expressing the FRET sensors were washed to substitute the DMEM with the experimental buffer. Basal FRET ratio was measured in 90 µL buffer. Subsequently, 10 µL of 10-fold ligand solution or buffer (negative control) was applied to each well and the stimulated FRET ratio was recorded. All FRET experiments were conducted at 37 °C with a Synergy Neo2 plate reader (BioTEK) equipped with 420/50 nm excitation and 485/20 nm emission filters for CFP. Acceptor emission of YFP, HaloTag® R110, HaloTag® diAcFAM, HaloTag® Oregon Green® and SNAP-cell 505-Star were detected with a 540/25 nm (FRET) filter. To measure the emission of HaloTag® TMR-Direct and SNAP-cell TMR-Star a 590/35 nm filter was used. Emission of HaloTag® NanoBRET 618 and SNAP-cell 647SiR were detected with a 620/15 nm and 680/20 nm filter, respectively. Fifty excitation flashes were applied per data point.

### BRET measurements

Cells transiently or stably expressing the BRET-biosensors were washed to substitute DMEM with the experimental buffer and incubated with substrate (90 µL of 1:1000 for β_2_AR_Nluc/Halo**618**_ and PTHR1 _Nluc/Halo**618**_; 1:4000 for α_2A_AR _Nluc/Halo**618**_) for 2–5 min at 37 °C to allow for substrate diffusion and the basal BRET ratio was measured. Following this, 10 µL of 10-fold ligand solution or buffer was applied to each well and the stimulated BRET ratio was recorded. To reduce the fluctuation of the BRET ratio in *Z*-factor experiments, seven individual BRET ratios within 5 min were measured and averaged before and after ligand addition.

BRET experiments were performed at 37 °C with a GloMAX Discover (Promega) or Synergy Neo2 (BioTEK) plate reader equipped with a 460/40 nm filter to select the NanoLuc emission. For cpVenus173, HaloTag® R110, HaloTag® diAcFAM, HaloTag® Oregon Green® and SNAP-cell 505-Star a 520/20 nm (BRET) filter was used to select the acceptor emission peaks. TagRFP, HaloTag® TMR-Direct and SNAP-cell TMR-Star emissions were detected with a 530 nm long pass filter. For HaloTag® NanoBRET 618 a 620/20 nm filter was used and a 600 nm long pass filter was applied for the BRET acceptors mCherry and SNAP-cell 647SiR. The integration time per data point was set to 0.3 s.

Experiments with higher temporal resolution were performed employing the Synergy Neo2 (BioTEK) plate reader, which is equipped with injectors and has a faster acquisition time. Data were acquired in well-mode, the acquisition interval was set to 1 s and the integration time to 0.3 s. After acquisition of baseline for 180 s, 10 µL of solution with or without ligand (buffer control) were injected with a speed of 225 µL s^−1^ (delivery time = 44 ms) and the signal was recorded for 180–360 s.

### Receptor staining

Cells were co-transfected with FRET-based sensors to monitor downstream signaling and the wild type or the BRET-biosensor receptor as described above. Staining of the plasma membrane portion of the receptors was evaluated using a cell-impermeable anti HA-tag conjugated with AlexaFluor594 (Anti-HA-AlexaFluor594 ThermoFischer) or Anti-Flag® M2 conjugated with Cy3 (Anti-Flag® M2 Cy3, Sigma). The fluorescent antibodies were diluted in DMEM to a concentration of 10 µg mL^−1^ and incubated for 1 h at 37 °C in the 96-well plates. Subsequently, cells were rinsed three times and incubated additional 30 min with fresh DMEM.

Subsequently, the emission intensity of HEK-TSA cells were measured using the Synergy Neo2 plate reader. Therefore, cells stained with Anti-Flag® M2 Cy3 (β_2_AR_NLuc/Halo_ or β_2_AR) were excited using a 540/20 nm excitation and the emission intensity was recorded using 590/35 nm emission filter. Fluorescence intensities of HEK-TSA cells stained with Anti-HA-AlexaFluor594 were measured using a 590/20 nm (excitation)–620/15 nm (emission) filter combination.

### Expression levels of Gα_i2_-FRET and H187-EPAC-FRET sensor

The Synergy Neo2 plate reader was employed to assess the expression levels of the downstream sensors (Gα_i2_-FRET and H187-EPAC-FRET sensor). Therefore, the FRET acceptors (cpVenus173 and tandem cpVenus173) were directly excited using a 500/20 nm excitation filter. Emission intensities were detected with a 540/20 nm filter.

### Membrane preparations

Membranes expressing wild-type α_2A_AR (α_2A_AR-wt) were harvested from HEK-TSA cells grown in 15 cm dishes, 48 h after transfection. Membranes expressing the BRET-based α_2A_AR sensor (α_2A_AR_Nluc/Halo_) were obtained from HEK293 stably expressing the sensor. Cells were detached from the dishes with a cell scraper and suspended in Tris buffer (5 mM Tris, 2 mM EDTA, pH 7.4). After centrifugation for 10 min at 1000 × *g*, cells were re-suspended in buffer 1 (20 mM HEPES, 10 mM EDTA, PBS, pH 7.4) and homogenized using twice Ultraturax for 15 s. The suspension was centrifuged for 10 min at 3200 × *g*. The resulting supernatant was further centrifuged for 45 min at 37,000 × *g* and 4 °C. The pellet was resuspended and the last two centrifugation steps were repeated. The pellet was then suspended in binding buffer (50 mM Tris, 100 mM NaCl, 3 mM MgCl_2_, pH 7.4) and the amount of total membrane protein was measured using the Pierce BCA Protein Assay Kit according to the manufacturer’s instructions.

### Radioligand binding

Total radioligand binding was assessed by incubating 5 µg of membrane protein with different concentrations (0.04–12 nM) of the antagonist α_2A_AR radioligand [^3^H]RX821002. To define unspecific binding, 20 µM phentolamine was added. Competition binding was performed by incubating 2 µg membrane protein with 0.3–2.0 nM [^3^H]RX821002 and increasing concentrations of the different α_2A_AR ligands in the presence (=low affinity state for agonists) and absence (=high-affinity state for agonists) of 10 µM GTP. Following incubation for 1 h at room temperature, membranes were transferred to Millipore glass-fiber filters via vacuum filtration. These filters were incubated with scintillation cocktail and membrane-bound radioactivity was measured with a scintillation counter.

### Data analysis and statistics

FRET and BRET ratios before (Ratio_basal_) and after ligand or buffer application (Ratio_stim_) were defined as acceptor emission/donor emission and corrected for donor bleedthrough into the acceptor channel by subtracting the averaged ratio of the unlabeled control (UC). For cells expressing biosensors with a fluorescent protein as acceptor, the averaged UC ratio of the analogous HaloTag construct was considered for bleedthrough correction.

To quantify the ligand induced conformational change, ∆FRET or ∆BRET was calculated for each well as a percent over basal (((Ratio_stim_ − Ratio_basal_)/Ratio_basal_) × 100) and subtracted by the averaged ∆FRET or ∆BRET of buffer.

*Z*-factors expressing the high-throughput suitability were calculated with the following equation:

$$Z = 1 - \frac{{\left( {3\sigma _S + 3\sigma _C} \right)}}{{\left( {{\mathrm{\mu }}_S - {\mathrm{\mu }}_C} \right)}}$$

where *σ*_s_ and *σ*_c_ are the standard deviations of ∆FRET or ∆BRET. *μ*_S_ and *μ*_C_ express the mean of ∆FRET or ∆BRET values of positive and negative control, respectively. If the positive control induced a decrease in the energy transfer (negative ∆RET as for α_2A_AR_CFP/YFP_, β_2_AR_NanoLuc/Halo**618**_, PTHR1_NanoLuc/Halo**618**_, PTHR1_CFP/YFP_) the denominator in equation is inverted (*μ*_C_ − *μ*_S_). As a positive control, we defined epinephrine for the α_2A_AR- and β_2_AR-sensors and PTH(1-34) for PTHR1 sensors. Buffer was used as a negative control in all *Z* factor experiments.

For simplicity, all agonist-induced RET changes were consistently plotted as ascending curves or bars. Therefore, *y*-axes in all figures were inverted if agonists for the respective biosensor induced a reduction of the ratio.

Data were analyzed using Prism 5.0 software (GraphPad) and expressed as mean ± s.e.m. Data from concentration–response experiments were fitted using a mono-exponential curve four-parameter fit. Radioactivity values from binding experiments were analyzed using a one-site fitting model if GTP was added prior the experiment. Data from competition-binding experiments without exogenously added GTP were first analyzed for the statistically preferred fitting model applying extra-sum-of squares F-test comparing a one-component vs. two-component fit. Superiority of the two-component model was confirmed for all agonists (partial or full) tested. The two-component fit was then conducted with the fraction of the high-affinity component (*R*_H_) fixed to 0.58 which is the mean *R*_H_ of all data where this model was applied. Statistical differences were evaluated using one-way ANOVA test followed by Bonferroni multiple comparison, Student’s *t*-test or extra-sum-of squares F-test. Differences were considered significant for values of *p* < 0.05.

### Data availability

The datasets generated during and/or analysed during the current study are available at the homepage of the Institute of Pharmacology and Toxicology http://www.pharmakologie.uni-wuerzburg.de/fileadmin/03250100/user_upload/Schihada_et_al._CommsBio_2018_-_Figure_1-3.zip

## Electronic supplementary material

Supplementary Information
