# Supplementary material for: A universal bioluminescence resonance energy transfer sensor design enables high-sensitivity screening of GPCR activation dynamics
Source: Commun Biol. 2018 Aug 7;1:105. doi: 10.1038/s42003-018-0072-0 (PMC6123785; doi:10.1038/s42003-018-0072-0)
Supplement: Supplementary file 1 — Supplementary Information [file 42003_2018_72_MOESM1_ESM.pdf]

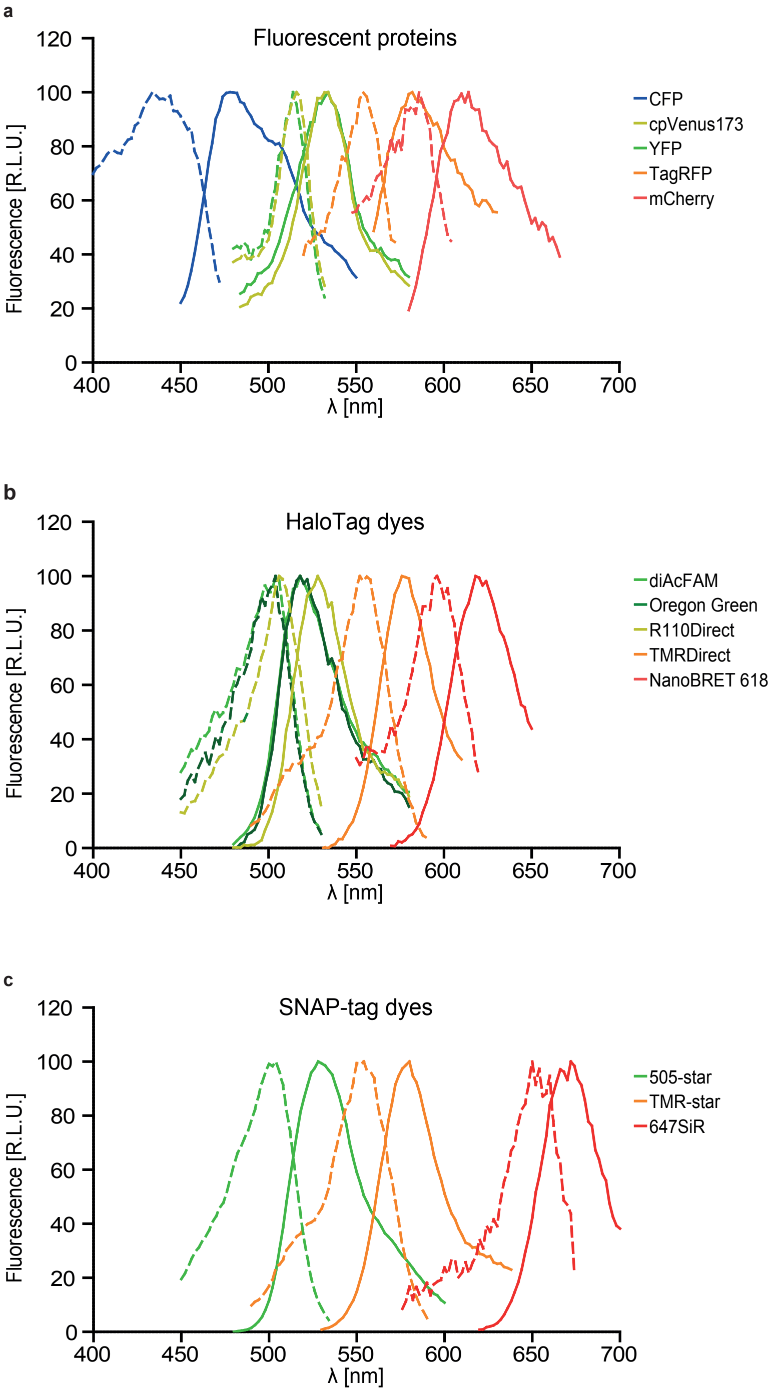

Supplementary Figure 1. Excitation and emission spectra of fluorescent proteins and dyes employed in the study. HEK-TSA cells were transiently transfected with α_{2A}AR fused to different tags. The excitation (dotted line) and emission (filled line) spectra were recorded of (**a**) fluorescent proteins and the self-labeling protein tags (**b**) SNAP and (**c**) Halo-tag after labeling (each N=1).

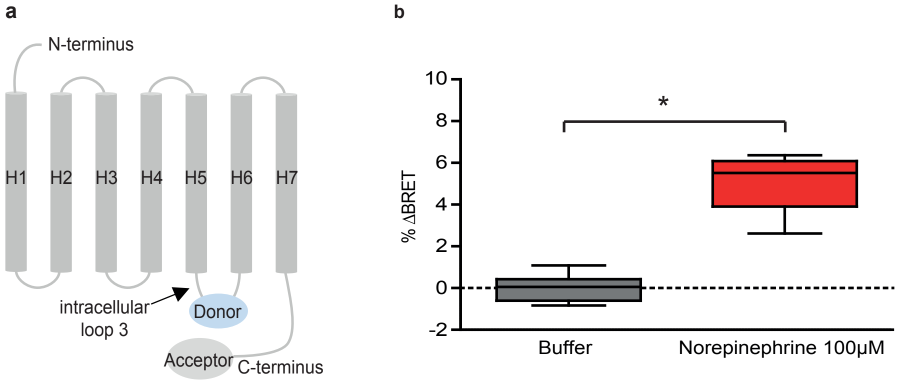

Supplementary Figure 2. Effect on BRET amplitude caused by the inversion of the positions of donor and acceptor in the α_{2A}AR-biosensor. Cells were transiently transfected with the inverted α_{2A}AR BRET sensor version (**a**) Schematic of the biosensor (**b**) Amplitude of norepinephrine-induced change in BRET normalized for buffer. Data show box and whisker plots of 4 independent experiments. Difference was analyzed by Student's t-test. *$p \leq 0{,}05$ versus buffer.

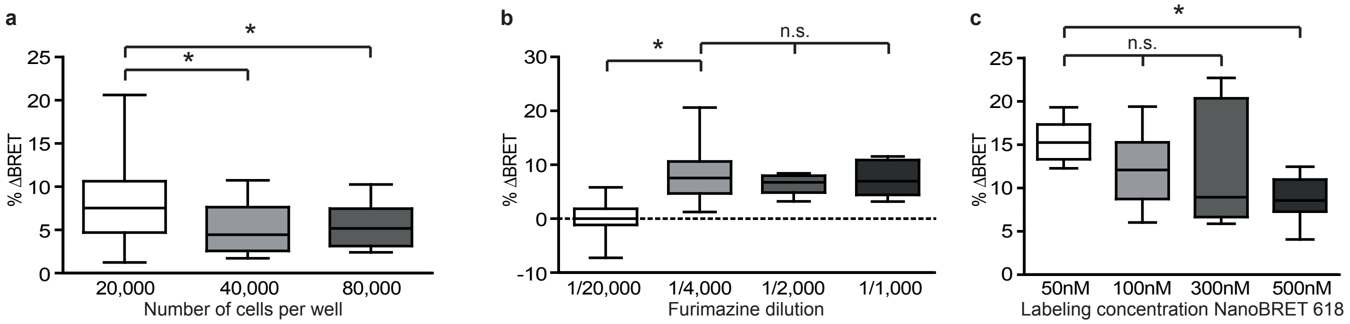

Supplementary Figure 3. Optimization of the BRET assay. (**a**) Increasing number of transiently transfected cells ($\alpha_{2A}AR_{Nluc/Halo}$) were seeded in 96-well plates and the 100 μM norepinephrine-induced BRET changes were evaluated for a given dilution of substrate (1/4,000) and concentration of HaloTag dye (100 nM). (**b**) 20,000 cells transiently expressing the $\alpha_{2A}AR_{Nluc/Halo}$ biosensor were plated per well and after labelling with the HaloTag618 dye (100 nM) the 100μM norepinephrine-induced BRET changes as a function of the substrate dilution (furimazine) were evaluated. (**c**) Cells stably expressing the $\alpha_{2A}AR_{Nluc/Halo}$ biosensor were plated in a 96-well plate (20,000 cells/well) and the effect of increasing concentrations of the fluorescent dye on the 100μM norepinephrine-induced BRET amplitude was estimated for a given substrate dilution (1/4,000). Data show box and whisker plots of 3 independent experiments. Difference was analyzed by two-way ANOVA followed by Bonferroni post hoc test. *$p \leq 0{,}05$ versus 20,000 cells (a); 1/4,000 furimazine dilution (b); 50nM NanoBRET618 (c)

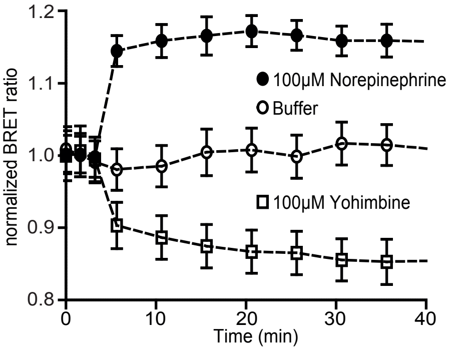

Supplementary Figure 4. Stability of the BRET change over time. Normalized BRET ratio of HEK293 cells stably expressing the $\alpha_{2A}AR_{Nluc/Halo}$ biosensor were treated with Norepinephrine (N=3), buffer (N=4) and Yohimbine (N=4). Data show mean±s.e.m.

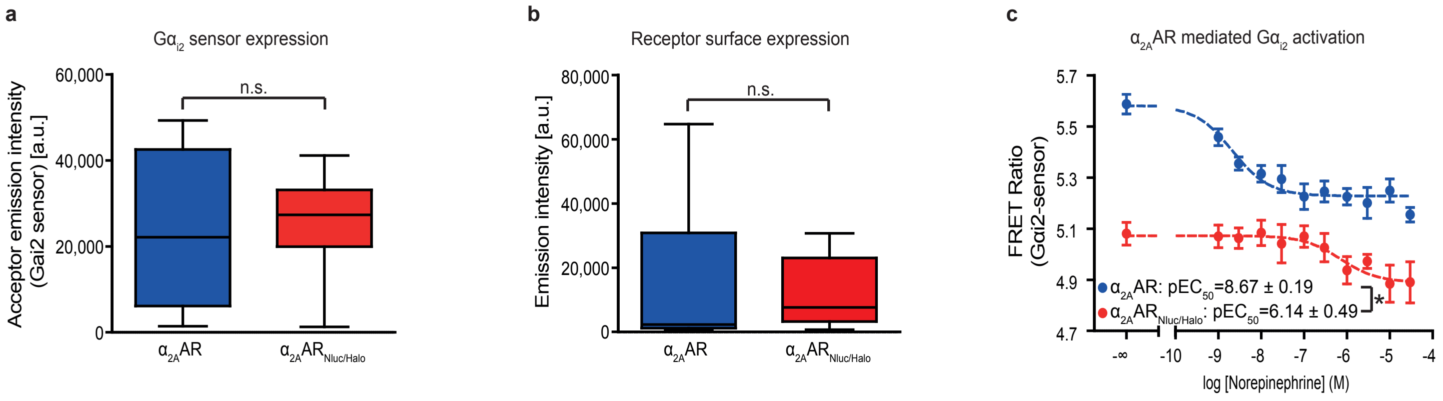

Supplementary Figure 5. Activation of downstream $G\alpha_{i2}$ is preserved for the $\alpha_{2A}AR_{Nluc/Halo}$ BRET-biosensor. G protein activation was evaluated in 96-well plates using the $G\alpha_{i2}$ FRET-sensor transiently co-transfected with $\alpha_{2A}AR$ wild-type receptor (blue) or with the biosensor $\alpha_{2A}AR_{Nluc/Halo}$ (red) both possessing an HA-tag at the N-terminus. (**a**) $G\alpha_{i2}$ expression: emission of $G\alpha_{i2}$ FRET acceptor upon direct excitation. (**b**) $\alpha_{2A}AR$ expression: emission of the fluorescent anti HA-tag antibody (Alexa-Fluor594-conjugated anti-HA). (**c**) FRET ratios upon stimulation with increasing concentrations of norepinephrine fitted to a single component concentration-response curve. Data show box and whisker plots (a,b) or mean±s.e.m. (c) of 3 independent experiments. In whisker plots, difference was analyzed by Student‘s t-test and Extra-sum-of squares F-test was applied to test for statistical difference between the two EC_{50} values in (c). $^*p \leq 0{,}05$.

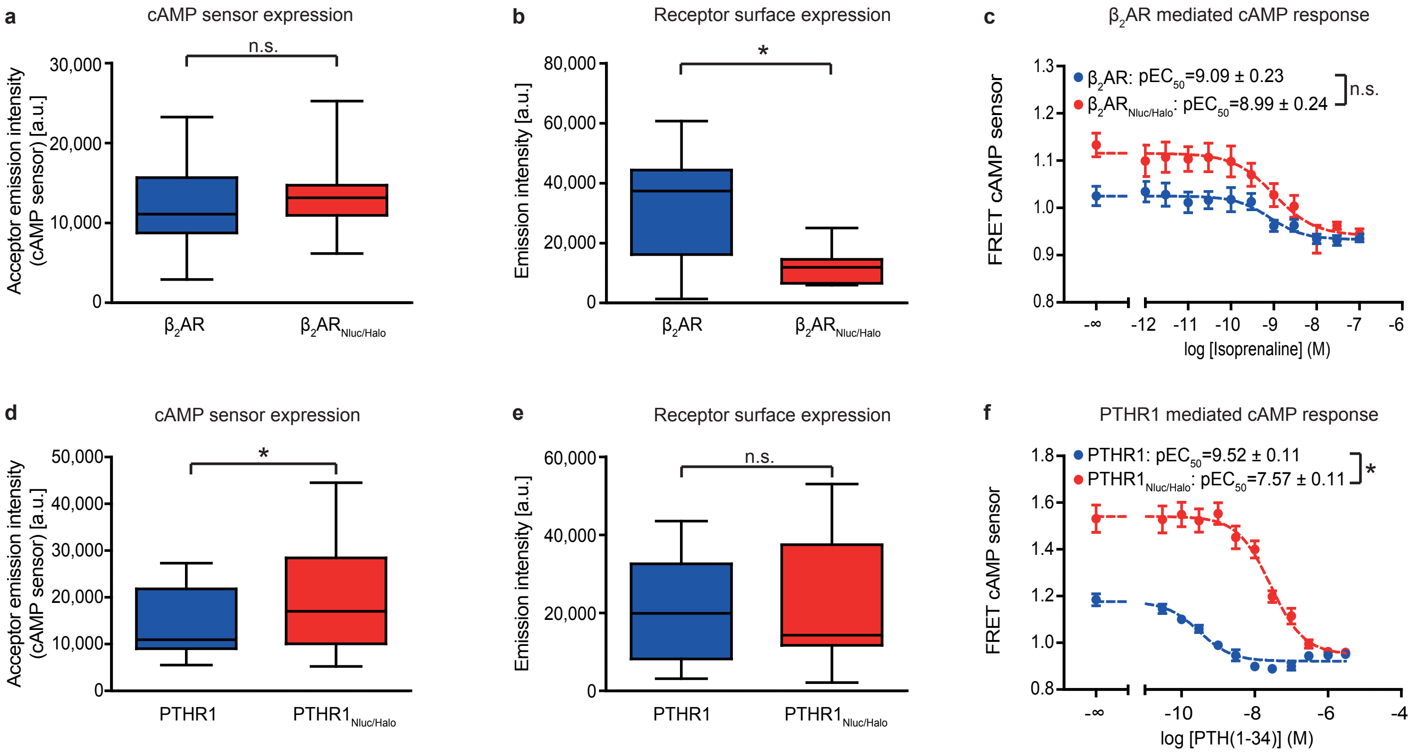

Supplementary Figure 6. cAMP signaling is preserved for the $\beta_2AR_{Nluc/Halo}$ and $PTHR1_{Nluc/Halo}$ BRET-biosensors. cAMP accumulation was evaluated in 96-well plates using the FRET-based H187-EPAC sensor transiently co-transfected with β_2AR and PTHR1 wild-type receptors (blue) or with the biosensors $\beta_2AR_{Nluc/Halo}$ or $PTHR1_{Nluc/Halo}$ (red). (**a, d**) H187-EPAC expression: emission of the cAMP FRET acceptor upon direct excitation. (**b, e**) Receptor expression: emission of the fluorescent Cy3-conjugated anti-FLAG antibody against the epitope on β_2AR (**b**) or anti-HA antibody against the epitope on PTHR1 (**e**). (**c, f**) Concentration-response curves obtained upon stimulation with increasing concentration of isoprenaline (**c**) or PTH(1-34) (**f**). FRET ratios are fitted to a mono-exponential concentration-response curve. Data show box and whisker plots (a,b,d,e) or mean±s.e.m. (c,f) of 3 independent experiments. In whisker plots, difference was analyzed by Student‘s t-test and Extra-sum-of squares F-test was applied to test for statistical difference between the two EC_{50} values in (c) and (f). *$p \leq 0,05$.

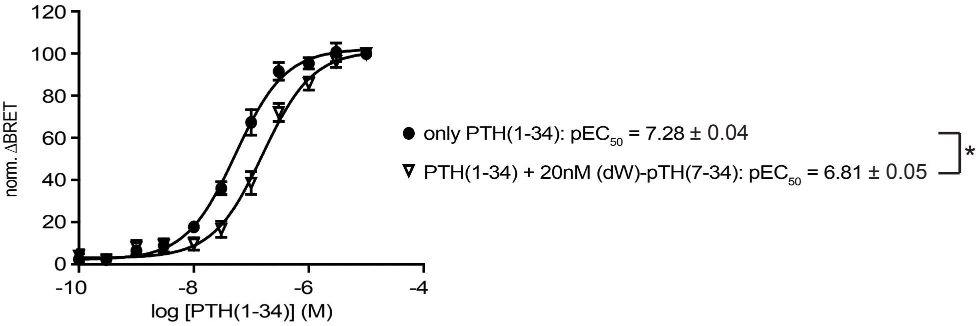

Supplementary Figure 7. Antagonistic effect of (dW)-PTH(7-34) on PTHR ΔBRET signals. Cells transiently expressing the $PTHR1_{Nluc/Halo}$ BRET-sensor were labeled and plated as described earlier. BRET amplitudes in presence of the full agonist PTH(1-34) alone (N=3) or after 60 minutes pre-treatment with the antagonist (20 nM) (N=4) were measured and fitted to a sigmoidal concentration-response curve. Data show mean±s.e.m. Extra-sum-of squares F-test was applied to test for statistical difference between the two EC_{50} values. *$p \leq 0,05$.

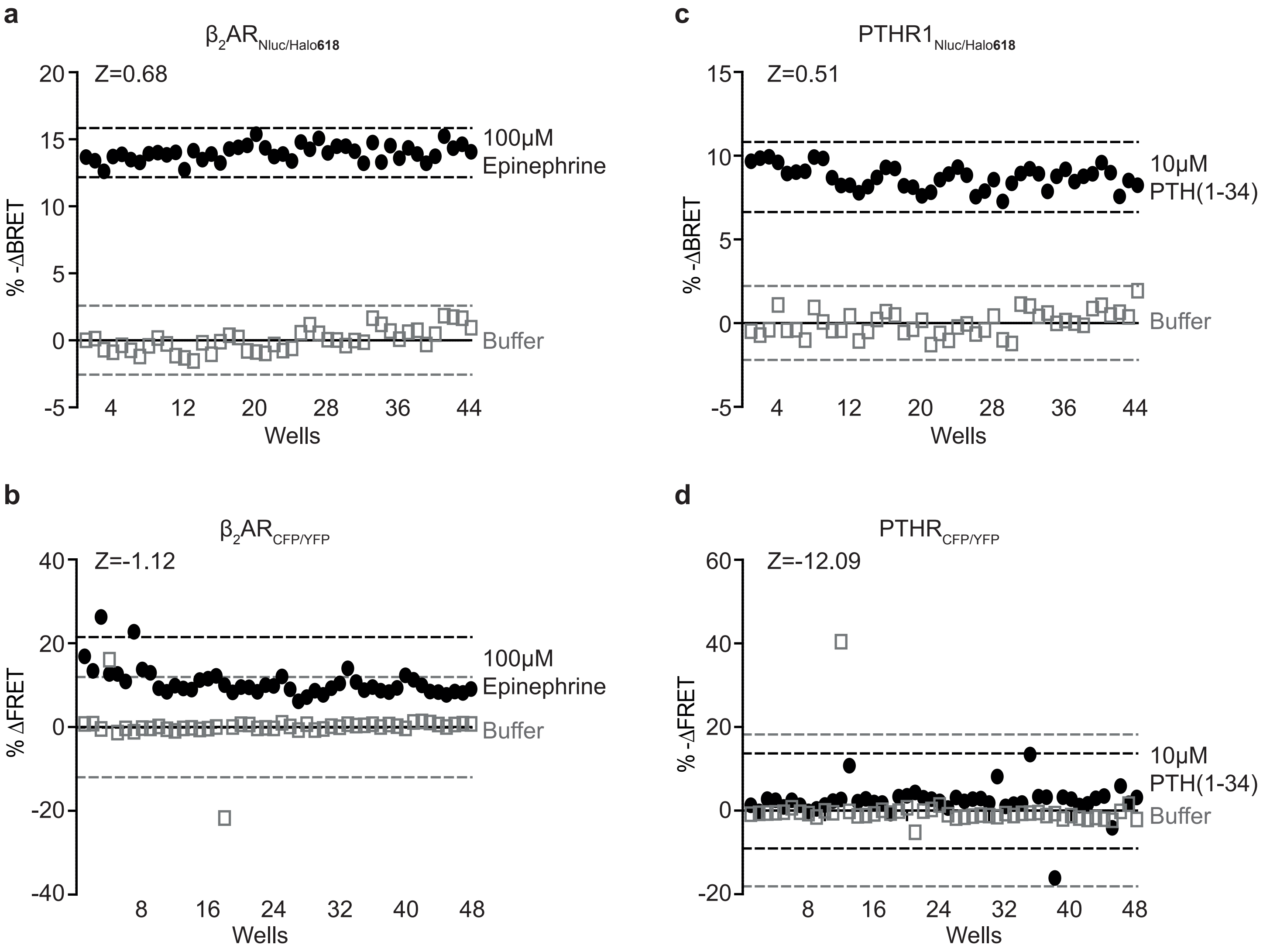

Supplementary Figure 8. Measurement of Z-factors for FRET and BRET based biosensors.Cells expressing the $FRET_{CFP/YFP}$ or $BRET_{Nluc/Halo}$ versions of the β_2AR and PTHR1 biosensors were transfected (for FRET biosensor), plated and labeled as described earlier in 96-well plates. Shown are representative experiments. Each data point expresses the ΔFRET or ΔBRET value of an individual well upon 100 μM epinephrine or buffer (**a,b**) and PTH(1-34) (10μM PTH(1-34)) or buffer (**c,d**) stimulation.

Target	Ligand	Activity*
α2AR	(-)-Epinephrine	Full agonist
	(-)-Norepinephrine	Full agonist
	UK 14,304	Full agonist / Partial agonist[1]
	Dopamine	Partial agonist[2]
	Oxymetazoline	Partial agonist
	Octopamine	Partial agonist[2]
	Clonidine	Partial agonist
	Phentolamine	Antagonist
	Tyramine	Antagonist[3]
	Yohimbine	Antagonist / Inverse agonist[4]
β2AR	(-)-Epinephrine	Full agonist
	Isoprenaline	Full agonist
	Salmeterol	Full Agonist
	Formoterol	Agonist
	(-)-Norepinephrine	Agonist
	Terbutaline	Partial agonist
	Salbutamol	Partial agonist
	Labetalol	Antagonist
	Carvedilol	Antagonist
	Metoprolol	Antagonist
	Propranolol	Antagonist
	ICI 118.551	Inverse agonist
PTH1R	PTH(1-34)	Full agonist
	PTHrP(1-34)	Full agonist
	PTH(1-31)	Agonist[5]
	(dW)-PTH(7-34)	Antagonist
	PTH(7-34)	Antagonist[5]
	PTH(3-34)	Antagonist[5]

*: If no publication is cited, ligand activity corresponds to the IUPHAR ligand classification database. (http://www.guidetopharmacology.org/). Only for compounds where no entry for the specific target could be found in the IUPHAR database, primary literature has been cited as a reference.

Supplementary Table 1. Expected activities of applied compounds

**Ligand**	**α_{2A}AR wild-type**<br>**pk_i ± s.e.m. (N)**	**$\alpha_{2A}AR_{Nluc/Halo}$**<br>**$pk_i$ ± s.e.m. (N)**
Epinephrine	4.83 ± 0.05 (3)	5.17 ± 0.16 (4)
Norepinephrine	4.57 ± 0.10 (3)	4.55 ± 0.14 (6)
UK 14,304	5.57 ± 0.25 (3)	5.42 ± 0.02 (4)
Oxymetazoline	6.47 ± 0.07 (3)	6.56 ± 0.08 (4)
Octopamine	3.66 ± 0.11 (4)	3.99 ± 0.09 (4)
Clonidine	5.81 ± 0.05 (3)	5.88 ± 0.09 (4)
Phentolamine	6.31 ± 0.09 (3)	6.87 ± 0.19 (4)
Tyramine	3.09 ± 0.14 (3)	3.87 ± 0.01 (3)
Yohimbine	6.09 ± 0.42 (4)	6.60 ± 0.10 (4)
	**pk_D ± s.e.m. (N)**	**pk_D ± s.e.m. (N)**
[^{3}H]RX821002	9.17 ± 0.05 (3)	9.19 ± 0.05 (3)

Supplementary Table 2. Comparison of ligand binding to the low affinity states of α_{2A}AR-wild-type versus $\alpha_{2A}AR_{Nluc/Halo}$ biosensor.

Ligand	$\alpha_{2A}AR_{Nluc/Halo}$ pk$_i$ ± s.e.m. (N)		BRET assay $\alpha_{2A}AR_{Nluc/Halo}$ pEC$_{50}$ ± s.e.m. (N)
	**High affinity state**	**Low affinity state**	
Epinephrine	5.79 ± 0.15 (3)	5.17 ± 0.16 (4)	6.40 ± 0.10 (4)
Norepinephrine	5.26 ± 0.08 (3)	4.55 ± 0.14 (6)	5.70 ± 0.09 (6)
UK 14,304	6.62 ± 0.20 (3)	5.42 ± 0.02 (4)	7.23 ± 0.23 (3)
Oxymetazoline	6.82 ± 0.21 (3)	6.56 ± 0.08 (4)	6.24 ± 0.15 (4)
Octopamine	4.25 ± 0.02 (3)	3.99 ± 0.09 (4)	4.38 ± 0.14 (4)
Clonidine	6.77 ± 0.03 (3)	5.88 ± 0.09 (4)	6.81 ± 0.13 (4)
Phentolamine	n.d.	6.87 ± 0.19 (4)	7.17 ± 0.14 (5)
Tyramine	n.d.	3.87 ± 0.01 (3)	4.35 ± 0.12 (4)
Yohimbine	n.d.	6.60 ± 0.10 (4)	6.61 ± 0.13 (7)

Supplementary Table 3. pEC$_{50}$ of α_{2A}AR ligands in the GPCR BRET sensor assay compared to ligand binding data of $\alpha_{2A}AR_{Nluc/Halo}$.

Ligand	pEC_{50} ± s.e.m.<br>BRET sensor	pk_i (95% CI)<br>radioligand binding
Epinephrine	6.45 ± 0.14	6.13 (5.98 – 6.29)[6]
Carvedilol	n.a.	8.96 (8.80 – 9.10)[6]
ICI 118,551	9.57 ± 0.16	9.15 (8.96 – 9.40)[6]

Supplementary Table 4. pEC_{50} of β_2AR ligands in the GPCR BRET sensor assay compared to literature data.

Ligand	pEC_{50} ± s.e.m. BRET sensor	pki ± s.e.m. radioligand binding
PTH (1-34)	7.28 ± 0.04	7.41 ± 0.04[7]
PTHrP (1-34)	7.69 ± 0.08	7.82 ± 0.15 / 8.13 ± 0.33[8]
PTH (3-34)	6.63 ± 0.49	n.a.

Supplementary Table 5. pEC_{50} of PTH1R ligands in the GPCR BRET sensor assay compared to radioligand binding.

Supplementary References:

1. Sungkaworn, T. et al. Single-molecule imaging reveals receptor-G protein interactions at cell surface hot spots. *Nature* **550**, 543-547 (2017).
2. Peltonen, J.M. et al. Molecular mechanisms of ligand-receptor interactions in transmembrane domain V of the alpha2A-adrenoceptor. *Br J Pharmacol* **140**, 347-358 (2003).
3. Nikolaev, V.O., Hoffmann, C., Bunemann, M., Lohse, M.J. & Vilardaga, J.P. Molecular basis of partial agonism at the neurotransmitter alpha2A-adrenergic receptor and Gi-protein heterotrimer. *The Journal of biological chemistry* **281**, 24506-24511 (2006).
4. Wade, S.M., Lan, K., Moore, D.J. & Neubig, R.R. Inverse agonist activity at the alpha(2A)-adrenergic receptor. *Molecular pharmacology* **59**, 532-542 (2001).
5. Appleton, K.M. et al. Biasing the parathyroid hormone receptor: relating in vitro ligand efficacy to in vivo biological activity. *Methods Enzymol* **522**, 229-262 (2013).
6. Hoffmann, C., Leitz, M.R., Oberdorf-Maass, S., Lohse, M.J. & Klotz, K.N. Comparative pharmacology of human beta-adrenergic receptor subtypes--characterization of stably transfected receptors in CHO cells. *Naunyn Schmiedebergs Arch Pharmacol* **369**, 151-159 (2004).
7. Gardella, T.J., Luck, M.D., Jensen, G.S., Usdin, T.B. & Juppner, H. Converting parathyroid hormone-related peptide (PTHrP) into a potent PTH-2 receptor agonist. *The Journal of biological chemistry* **271**, 19888-19893 (1996).
8. Gardella, T.J. et al. Parathyroid hormone (PTH)-PTH-related peptide hybrid peptides reveal functional interactions between the 1-14 and 15-34 domains of the ligand. *The Journal of biological chemistry* **270**, 6584-6588 (1995).
